# A Reduction in ADAM17 Expression Is Involved in the Protective Effect of the PPAR-*α* Activator Fenofibrate on Pressure Overload-Induced Cardiac Hypertrophy

**DOI:** 10.1155/2018/7916953

**Published:** 2018-07-19

**Authors:** Si-Yu Zeng, Hui-Qin Lu, Qiu-Jiang Yan, Jian Zou

**Affiliations:** ^1^Department of Drug Clinical Trials, Guangdong Second Provincial General Hospital, Guangzhou 510317, China; ^2^Department of Cardiac & Thoracic Surgery, The Third Affiliated Hospital of Guangzhou Medical University, Guangzhou 510000, China; ^3^Department of Pharmacy, The People's Hospital of Pengzhou, Chengdu 611900, China

## Abstract

The peroxisome proliferator-activated receptor-*α* (PPAR-*α*) agonist fenofibrate ameliorates cardiac hypertrophy; however, its mechanism of action has not been completely determined. Our previous study indicated that a disintegrin and metalloproteinase-17 (ADAM17) is required for angiotensin II-induced cardiac hypertrophy. This study aimed to determine whether ADAM17 is involved in the protective action of fenofibrate against cardiac hypertrophy. Abdominal artery constriction- (AAC-) induced hypertensive rats were used to observe the effects of fenofibrate on cardiac hypertrophy and ADAM17 expression. Primary cardiomyocytes were pretreated with fenofibrate (10 *μ*M) for 1 hour before being stimulated with angiotensin II (100 nM) for another 24 hours. Fenofibrate reduced the ratios of left ventricular weight to body weight (LVW/BW) and heart weight to body weight (HW/BW), left ventricular anterior wall thickness (LVAW), left ventricular posterior wall thickness (LVPW), and ADAM17 mRNA and protein levels in left ventricle in AAC-induced hypertensive rats. Similarly,* in vitro* experiments showed that fenofibrate significantly attenuated angiotensin II-induced cardiac hypertrophy and diminished ADAM17 mRNA and protein levels in primary cardiomyocytes stimulated with angiotensin II. In summary, a reduction in ADAM17 expression is associated with the protective action of PPAR-*α* agonists against pressure overload-induced cardiac hypertrophy.

## 1. Introduction

Hypertension, a critical cardiovascular disease, is responsible for many disabilities and deaths worldwide. It is characterized by sustained pressure overload and concurrent development of pathological cardiac hypertrophy, which plays a critical role in the onset and development of chronic heart failure, the end stage of the cardiovascular event chain [1, 2]. The five-year-survival rate has been reported to be very low in symptomatic patients with chronic heart failure [3, 4]. Thus, improving pathological cardiac hypertrophy may prevent the progression from hypertension to chronic heart failure.

Pressure overload and cardiac hypertrophy share common inducers (e.g., endothelin and angiotensin II) that activate downstream matrix metalloproteinases (MMPs, such as MMP2 and MMP7) and a disintegrin and metalloproteinases (ADAMs, such as ADAM12 and ADAM17) via activating Gq protein-coupled receptors [2, 5–7]. Among these metalloproteinases, MMP2, MMP7, ADAM12, and ADAM17 are the most well studied in the context of the cardiovascular system. Previous studies have verified that ADAM17 lies on upstream of ADAM12 and MMP2 in the network of the metalloproteinase signaling pathway [2, 8], indicating that ADAM17 could be a key member of the metalloproteinases family. ADAM17 has essential functions in cell-cell interactions, signaling, and proteolysis of key cytokines, cytokine receptors, and other targets [9, 10]. Systemic ADAM17 knockdown was shown to ameliorate cardiac hypertrophy in angiotensin II-induced hypertensive mice and spontaneously hypertensive rats [7, 8]. Therefore, ADAM17 is a crucial factor that mediates pressure overload-induced cardiac hypertrophy.

PPAR-*α* is present in high levels in tissues with high energy demands that depend on the oxidation of mitochondrial fatty acids as a primary energy source, including the heart and liver [11]. Recently, PPAR-*α* activators have been evaluated as therapeutic agents to modulate cardiac hypertrophy alone or in conjunction with other agents [12–16]. However, it remains unclear whether ADAM17 participates in the protective effect of fenofibrate against cardiac hypertrophy.

In this research, we found that PPAR-*α* activation by fenofibrate ameliorated pressure overload-induced cardiac hypertrophy and reduced ADAM17 expression in left ventricular tissue in AAC-induced hypertensive rats. In cultured primary cardiomyocytes, PPAR-*α* activation inhibited angiotensin II-induced cardiac hypertrophy and decreased ADAM17 protein and mRNA levels. Our previous results showed that ADAM17 siRNA markedly attenuated angiotensin II-induced cardiac hypertrophy in primary cardiomyocytes [17]. Therefore, these lines of indirect evidence indicate that a decrease in ADAM17 expression is involved in the protective effect of fenofibrate on pressure overload-induced cardiac hypertrophy.

## 2. Materials and Methods

### 2.1. Animals

Animal protocols were performed according to the guidelines principles for the Care and Use of Laboratory Animals issued by National Institutes of Health of the United States. Further, the animal experiments were approved by the Medical Ethics Committees of Guangdong Second Provincial General Hospital. A total of 46 male Sprague-Dawley rats (about 200-250 g) were purchased from the Experimental Animals Center of Sun Yat-Sen University.

### 2.2. AAC-Induced Cardiac Hypertensive Model

After rats were anesthetized via intraperitoneal injection of 3% sodium pentobarbital (40mg /kg), midline celiotomy was performed to expose the abdominal artery just above the kidney artery. Aortic banding was carried out according to the method described by Irukayama-Tomobe Y [15]. AAC was performed in 36 rats, which were divided into the AAC rats group (vehicle, oral gavage), AAC+Fenofibrate (60mg/kg, oral gavage) group, and AAC+ Fenofibrate (120mg/kg, oral gavage) group. The 60 mg/kg and 120 mg/kg doses of fenofibrate in rats are two times and four times, respectively, as much as the equivalent dose in humans. Likewise, these same procedures were carried out in rats of the Sham group except for the binding of the abdominal artery. There were 10 rats in each group. Sustained AAC for 4 weeks caused no deaths in the rats even though there was about 15% mortality within 24 hours in rats subjected to AAC.

### 2.3. Echocardiography and Hemodynamic Measurements

Ultrasonic electrocardiograph images were obtained with a Vevo 2100 high-resolution* in vivo* microimaging system (Visual Sonics, Canada) after rats were anesthetized through inhalation of 2% enflurane as described previously; left ventricular anterior and posterior wall thickness (LVAW and LVPW) were measured and analysed [17, 18]. Next, aortic systolic pressure (AoSP), left ventricular systolic pressure (LVSP), and maximal rate of left ventricular pressure increase (dp/dtmax) and decrease (dp/dtmin) were measured using a BL-420S system (Chengdu Tai-Meng Technology Co., Ltd., China). Finally, heart mass index and left ventricular mass index were calculated separately.

### 2.4. Cell Culture

Primary cultures of ventricular cardiomyocytes were obtained from 2-day-old Sprague- Dawley rats as previously described [19].

### 2.5. Cellular Surface Area

After staining with tetramethylrhodamine- (TRITC-) labelled phalloidin, cardiomyocytes were used to evaluate cell surface area by automatically analysing the mean cell area of 40 visual fields using Cellomics/High Content Screening (Thermo Scientific, America) as previously described [17].

### 2.6. Real Time Quantitative PCR

RNA extraction and real time quantitative PCR were carried out as described previously [17]. For real time quantitative PCR, specific primers against ADAM17, BNP, and ANP were used and the GADPH gene was used as an inner control. The following premiers were used: ADAM17: 5′-GTGAGCAGTTTCT CGAACGC-3′ (forward primer) and 5′-AGCTTCTCAAGTCGCAGGTG-3′ (reverse primer); BNP: 5′-ATGCAGAAGCTGCTGGAGCTGATA-3′ (forward primer) and 5′-TTG TAGGGCCTTGGTCCTTTGAGA-3′ (reverse primer); ANP: 5′-GGAAGTCAACCCGTCTCA-3′ (forward primer) and 5′-AGCCCTCAGTTTGCTTTT-3′ (reverse primer); GADPH: 5′-ATCAA GAAGGTGGTGAAGCA-3′ (forward primer), 5′-AAGGTGGAAGAATGGGAGTTG-3′ (reverse primer).

### 2.7. Western Blotting

Protocols for western blotting were based on a previously reported method [17]. Antibodies included antibody against ADAM17 (Abcam, America), antibody against tubulin (Santa Cruz, America), and goat anti-rabbit lgGHRP (Affinity, America).

### 2.8. Statistical Analysis

Data are expressed as the mean ± SD. Results were analysed using an unpaired t-test between two groups and one-way ANOVA among at least three groups. Results were considered to be statistically significant when* p<0.05*.

## 3. Results

### 3.1. Fenofibrate Protected against Pressure Overload-Induced Cardiac Hypertrophy

**Figure 1:**
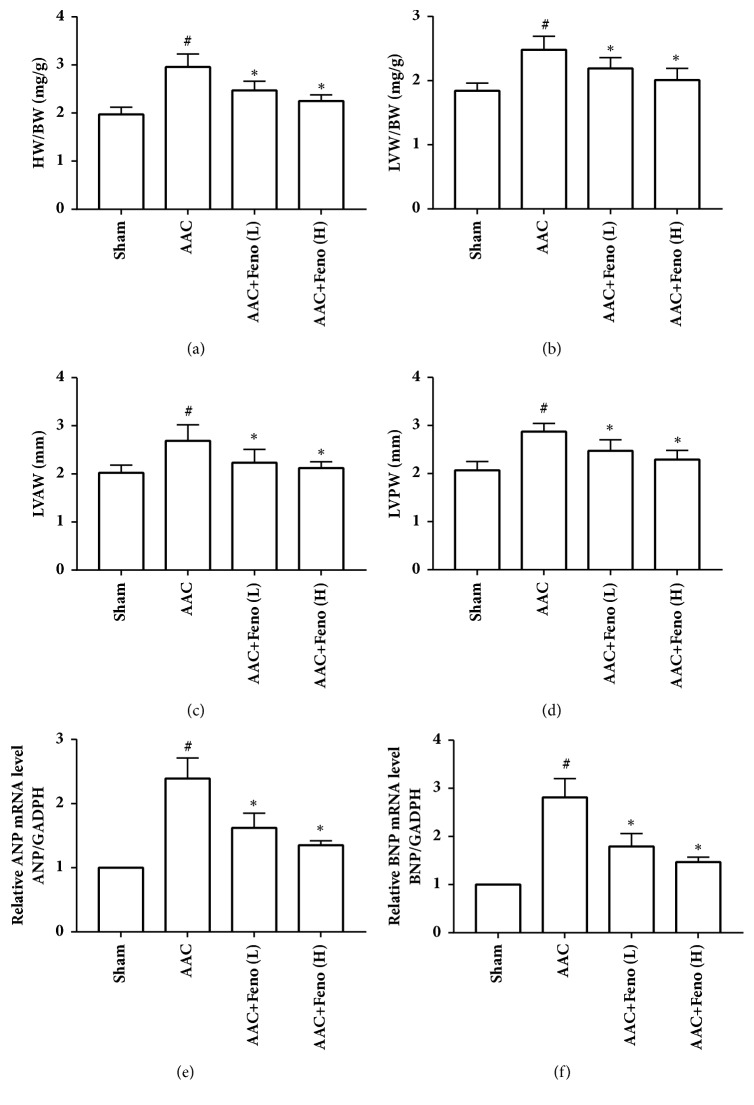
Fenofibrate inhibited pressure-overload-induced cardiac hypertrophy in abdominal artery constriction- (AAC-) induced hypertensive rats. (a) HW/BW; (b) LVW/BW; (c) LVAW; (d) LVPW; (e) ANP mRNA level; (f) BNP mRNA level. Feno represents fenofibrate; HW/BW represents ratio between heart weight and body weight; LVW/BW represent ratio between left ventricular weight and body weight; LVAW represents left ventricular anterior wall thickness; LVPW represents left ventricular posterior wall thickness; ^#^p<0.05 versus Sham group; *∗*p<0.05 versus AAC group, n=6 per group.

As shown in Table 1, there were significant increases in aortic systolic pressure [AoSP, 180.6 ±13.7 mm Hg versus 124.3±8.6 mm Hg] and left ventricular systolic pressure [LVSP, 184.4±11.4 mm Hg versus 134.3±9.8 mm Hg] in the AAC group compared with those in the Sham group, indicating the successful construction of an AAC-induced hypertensive model. Cardiac hypertrophy was commonly assessed by ventricular mass and ventricular wall thickness, indicated by HW/BW, LVW/BW, LVAW, and LVPW [20]. After treatment with a low or high dose of fenofibrate for 28 days, rats subjected to AAC showed weakened hypertrophy in the left ventricle supported by decreased HW/BW, LVW/BW, LVAW, LVPW, and mRNA levels of hypertrophic genes (ANP and BNP), although treatment with fenofibrate caused no significant change in LVSP (Figure 1 and Table 1). Thus, fenofibrate can inhibit pressure overload-induced cardiac hypertrophy.

### 3.2. Fenofibrate Reduced ADAM17 Expression in Left Ventricular Tissue from AAC-Induced Hypertensive Rats

**Figure 2:**
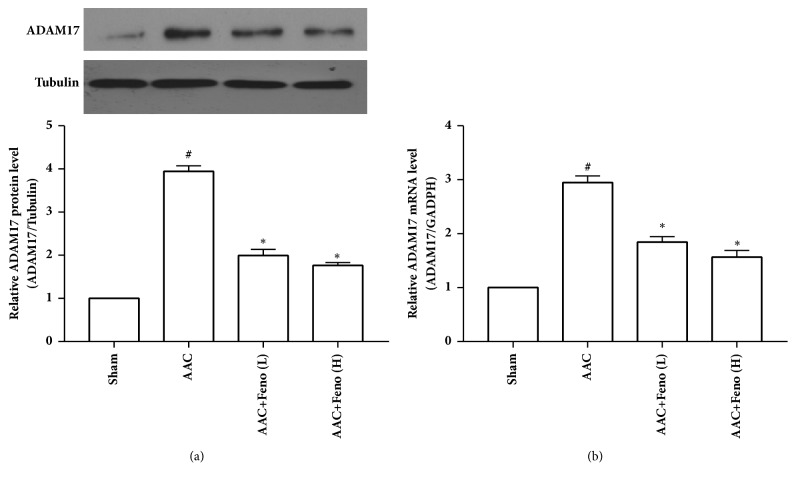
Fenofibrate reduced the expression of a disintegrin and metalloproteinase-17 (ADAM17) in left ventricular tissue from abdominal artery constriction- (AAC-) induced hypertensive rats. (a) ADAM17 protein level (n=4 independent experiments); (b) ADAM17 mRNA level (n=3 independent experiments). Feno represents fenofibrate; ^#^p<0.05 versus Sham group; *∗*p<0.05 versus AAC group.

As shown in Figures 2(a) and 2(b), ADAM17 protein and mRNA levels were significantly upregulated in the AAC group compared with those in the Sham group, whereas the low or high dose of fenofibrate significantly reduced ADAM17 protein and mRNA levels in left ventricular tissue from AAC-induced hypertensive rats. These findings showed that fenofibrate decreased ADAM17 expression in left ventricular tissue from AAC-induced hypertensive rats.

### 3.3. Fenofibrate Inhibited Angiotensin II-Induced Cardiac Hypertrophy in Cultured Primary Cardiomyocytes

**Figure 3:**
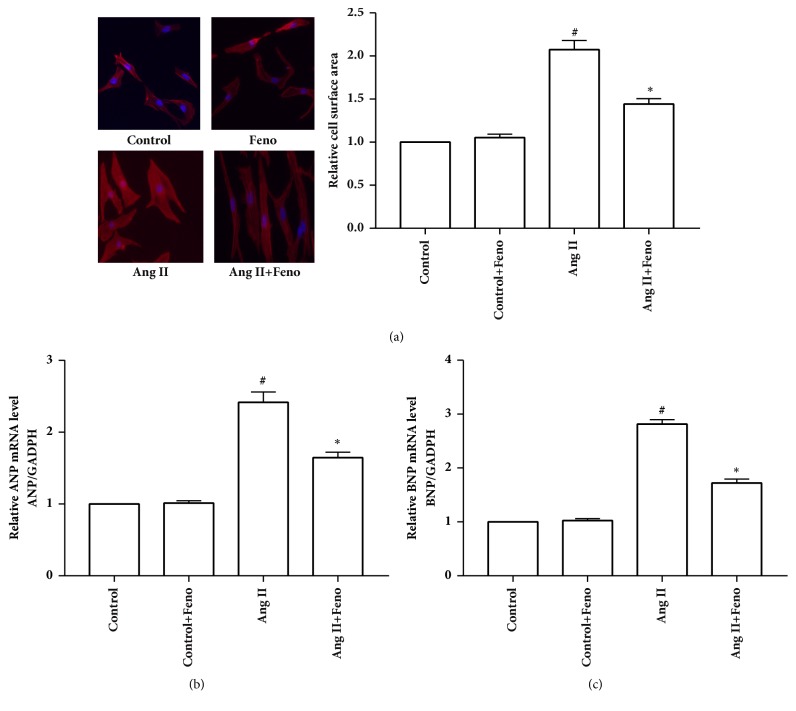
Fenofibrate attenuated angiotensin II-induced cardiac hypertrophy in cultured primary cardiomyocytes. After pretreated with fenofibrate (10 *μ*M) for 1 hour, cardiomyocytes were then stimulated with 100 nM angiotensin II for 24 hours. (a) Cell surface area; (b) ANP mRNA level; (c) BNP mRNA level. Ang II represents angiotensin II; Feno represents fenofibrate; ^#^p<0.05 versus control group; *∗*p<0.05 versus Ang II group. n=3 independent experiments.

Cardiac hypertrophy involves the reexpression of foetal genes, including *β*-myosin heavy chain (*β*-MHC), ANP, and BNP, and these foetal genes were commonly used as cell markers to diagnose cardiac hypertrophy [21]. Furthermore, cell surface area is also used to evaluate cardiac hypertrophy in cultured cardiac cells [22]. As shown in Figure 3, cell surface area and mRNA levels of hypertrophic markers (ANP and BNP) were markedly elevated in cardiomyocytes treated with 100nM angiotensin II for 24 hours compared with those in the control group, whereas treatment with fenofibrate reduced cell surface area and mRNA levels of ANP and BNP. These findings indicated that fenofibrate can alleviate angiotensin II-induced cardiac hypertrophy.

### 3.4. ADAM17 Mediated the Protective Action of Fenofibrate against Angiotensin II-Induced Cardiac Hypertrophy

**Figure 4:**
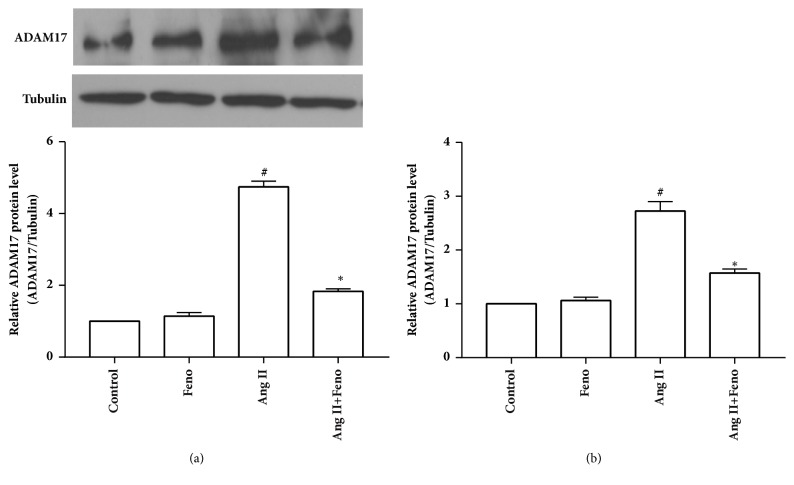
Fenofibrate reduced the expression of a disintegrin and metalloproteinase-17 (ADAM17) in primary cardiomyocytes stimulated with 100 nM angiotensin II for 24 hours. After pretreated with fenofibrate (10 *μ*M) for 1 hour, cardiomyocytes were then stimulated with 100 nM angiotensin II for 24 hours. (a) ADAM17 protein level (n=4 independent experiments); (b) ADAM17 mRNA level (n=3 independent experiments). Ang II represents angiotensin II; Feno represents fenofibrate; ^#^p<0.05 versus control group; *∗*p<0.05 versus Ang II group.

*In vitro* experiments showed that pretreatment with fenofibrate (10 *μ*M) inhibited angiotensin II-induced upregulation of ADAM17 expression (Figure 4). Our previous results revealed that ADAM17 siRNA could attenuate cardiac hypertrophy, as indicated by its effects on cell surface area and mRNA levels of hypertrophic genes (ANP and BNP) in primary cardiomyocytes stimulated with 100nM angiotensin II for 24 hours [17]. Together, these lines of indirect evidence showed that inhibition of ADAM17 modulated the protective action of fenofibrate against angiotensin II-induced cardiac hypertrophy.

## 4. Discussion

PPAR-*α* is a critical mediator of cardiac hypertrophy. An approximately 4-fold increase in PPAR-*α* expression in PPAR-*α* transgenic (Tg) mice compared with that in nontransgenic (NTg) littermates does not cause significant cardiac hypertrophy in PPAR-*α* Tg mice [23]. There was, however, a report of the induction of significant ventricular hypertrophy in normal mice in PPAR-*α* TG mice that express a higher PPAR-*α* levels compared with those of NTg littermates [24]. Nevertheless, Rana S et al. [25] reported that PPAR-*α* overexpression during pathological cardiac hypertrophy ameliorated cardiac hypertrophy and improved heart function. PPAR-*α* expression is downregulated in the failing human hearts [26] but increased in the hearts of diabetic mice induced by streptozotocin and patients with dilated cardiomyopathy [24, 27]. Moreover, the absence of PPAR-*α* results in a more pronounced hypertrophic growth response and cardiac dysfunction associated with the enhanced expression of inflammatory markers and extracellular matrix remodelling [28]. These findings suggest that a certain amount of PPAR-*α* expression and activity is required for maintaining heart morphology and function, but excessive or lower expression and activity levels of PPAR-*α* promote cardiac hypertrophy and heart dysfunction under physiological conditions, whereas PPAR-*α* overexpression attenuates cardiac hypertrophy under pathological conditions.

The modest activation of PPAR-*α* with its agonist fenofibrate represents a potentially pharmacological intervention against pressure overload-induced cardiac hypertrophy. The PPAR-*α* agonist fenofibrate is commonly applied to treat hyperglyceridaemia, hypercholesterolemia, and mixed dyslipidaemia [29, 30]. It has been reported that fenofibrate suppresses cardiac hypertrophy, fibrosis, and inflammation in pressure overloaded rats [12–16, 31, 32], in agreement with our results that fenofibrate inhibited pressure overload-induced cardiac hypertrophy in rats subjected to AAC.

The mechanisms by which fenofibrate inhibits pressure overload-induced cardiac hypertrophy are very complicated. Fenofibrate attenuates pressure overload-induced cardiac hypertrophy partly by inhibiting the binding of p65-NF*κ*B to NFATc4, the c-Jun NH2-terminal kinase pathway, ERK activation, and MMP2 activity, as well as increasing high mobility group box 1 (HMGB1) levels in nuclei [12, 15, 33, 34]. However, additional details are needed to improve our understanding about how PPAR-*α* agonist fenofibrate suppresses cardiac hypertrophy.

Activated ADAM17 promotes the shedding of substrates, including proinflammatory factors [such as tumour necrosis factor *α* (TNF-*α*)] and growth factors [such as epidermal growth factor (EGF) and heparin-binding EGF-like growth factor (HB-EGF)] [10], thus triggering a cascade of mitogen-activated protein kinase to advance cardiac hypertrophy via the transcriptional activation of immediate-early genes and foetal genes referred to as hypertrophic genes [8]. Our previous results also showed that ADAM17 siRNA inhibited angiotensin II-induced cardiac hypertrophy in primary cardiomyocytes. Temporal systemic ADAM17 deletion suppresses the increase in systolic blood pressure and levels of fasting glucose and lipid in mice fed with a high-fat diet, indicating that ADAM17 might participate in the balance between fatty acid uptake and utilization in the heart [35]. Fatty acid uptake/utilization mismatch in the heart leads to lipid accumulation that is related to initial cardiac hypertrophy [36]; however, it needs to elucidate whether ADAM17 mediates cardiac hypertrophy through lipids accumulation in the heart. In the present study, our findings indicated that fenofibrate alleviated cardiac hypertrophy and reduced ADAM17 expression in hypertrophic cardiomyocytes* in vivo* and* in vitro* experiments. Collectively, decreased ADAM17 expression is associated with the protective effect of the PPAR-*α* activator fenofibrate on pressure overload-induced cardiac hypertrophy.

## 5. Conclusion

In conclusion, our indirect evidence indicates that a decrease in ADAM17 expression is related to the beneficial role of PPAR-*α* activation in pressure overload-induced cardiac hypertrophy. These results may aid in improving our understanding about how fenofibrate inhibits cardiac hypertrophy, thereby providing additional evidence for preventing from cardiac hypertrophy with fenofibrate. However, further research is needed to elucidate whether and how ADAM17 mediates the protective effects of fenofibrate on cardiac hypertrophy in hypertensive rats overexpressing ADAM17 gene in combination with fenofibrate treatment.

## Figures and Tables

**Table 1:** The effect of fenofibrate on hemodynamic data in rats with abdominal artery constriction.

	Sham	AAC	AAC+Feno (L)	AAC+Feno (H)
AoSP (mmHg)	124.3±8.6	180.6±13.7^#^	178.6±14.1	176.1±10.4
LVSP (mmHg)	134.3± 9.8	184.4±11.4^#^	180.8±10.9	178.5±10.7
HR (bpm)	348.8±18.2	358.6±16.1	354.0±15.2	350.8±12.8
+dp/dt_max_ (mmHg/sec.)	4.98±0.28	3.37±0.61^#^	4.53±0.45^*∗*^	4.79±0.29^*∗*^
-dp/dt_max_ (mmHg/sec.)	4.84±0.35	3.08±0.37^#^	4.27±0.41^*∗*^	4.56±0.28^*∗*^

AoSP, aortic systolic pressure; LVSP, left ventricular systolic pressure; HR, heart rate; dp/dt_max_, maximal rate of left ventricular pressure increase; dp/dt_min_, maximal rate of left ventricular pressure decrease. n=6 for each group, values are mean ± SD. ^#^p<0.05 versus sham group, *∗*p<0.05 versus AAC group. Feno represents fenofibrate, n=6 for each group.

## Data Availability

The data used to support the findings of this study are available from the corresponding author upon request.

## Supplementary Materials

Supplementary Materials Figure S1: additional western blot protein bands. A: ADAM17 protein level in the left ventricle in abdominal artery constriction- (AAC-) induced hypertensive rats. B: ADAM17 protein level in cardiomyocytes stimulated with angiotensin II for 24 hours.
