# Supplementary material for: A Reduction in ADAM17 Expression Is Involved in the Protective Effect of the PPAR-α Activator Fenofibrate on Pressure Overload-Induced Cardiac Hypertrophy
Source: PPAR Res. 2018 Jul 19;2018:7916953. doi: 10.1155/2018/7916953 (PMC6076894; doi:10.1155/2018/7916953)
Supplement: Supplementary Materials — Figure S1: additional western blot protein bands. A: ADAM17 protein level in the left ventricle in abdominal artery constriction- (AAC-) induced hypertensive rats. B: ADAM17 protein level in cardiomyocytes stimulated with angiotensin II for 24 hours. [file 7916953.f1.docx]

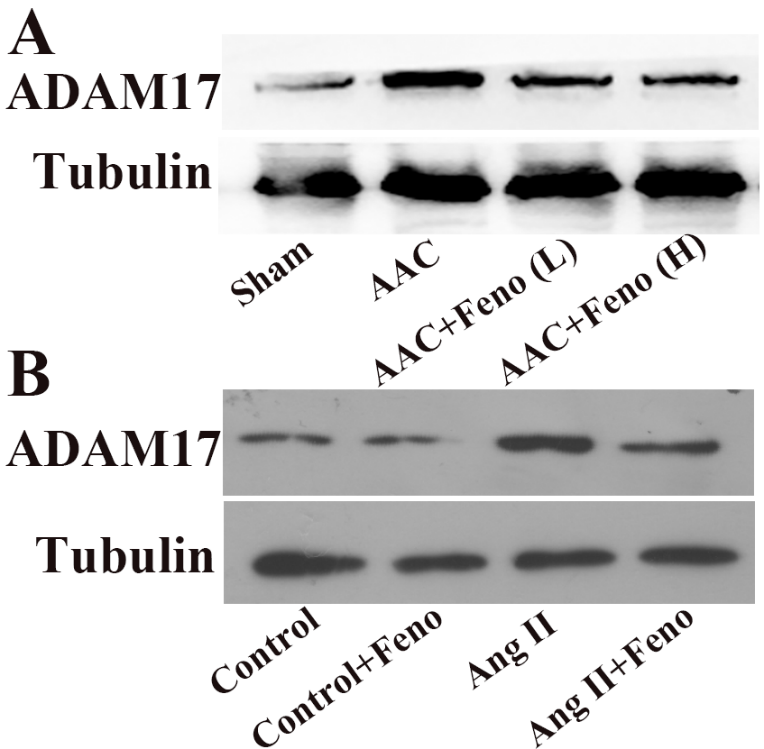


Figure S1 Additional protein bands of western blot. A: ADAM17 protein level in the left ventricle in abdominal artery constriction (AAC)-induced hypertensive rats. B: ADAM17 protein level in cultured cardiomyocytes stimulated with angiotensin II for 24 hours. Feno represents fenofibrate; Ang II represents angiotensin II.
